# A juvenile polyp on screening colonoscopy

**DOI:** 10.4322/acr.2021.414

**Published:** 2022-12-22

**Authors:** Spyridon Vrakas, Simone Ignatova, Giorgos Karapiperis, Sofia Kartsoli, Dimitrios Karapiperis

**Affiliations:** 1 Tzaneio General Hospital, Department of Gastroenterology, Athens, Greece; 2 University Hospital of Linkoping, Department of Pathology, Linkoping, Sweden; 3 Tzaneio General Hospital, ICU, Athens, Greece; 4 Vrinnevi General Hospital of Norrkoping, Department of Gastroenterology, Norrkoping, Sweden

**Keywords:** Colonic polyps, colonoscopy, colorectal neoplasms

The most common polyps in the large bowel are adenomas and serrated polyps. Diamond^1^ first described Juvenile polyps in 1939, and Helwig^2^ in 1946 reported what currently is coined as hamartomatous polyps. They are typically lobulated and pedunculated with erosions in the surface and appear cystic with dilated glands and inflammatory cells. They vary in size from a few millimeters to several centimeters. Juvenile polyps are rare in the general population, and the prevalence is difficult to determine. In the adult population, they comprise less than 1% of all detected polyps, and in children, over 90% of the polyp cases.^3^

The clinical management varies according to their localization and size. Polyps in the large bowel are detected during endoscopy and removed with polypectomy, in the stomach and duodenum during gastroscopy and in the small bowel with device-assisted enteroscopy.^4^

The risk of cancer when detecting one juvenile polyp is not clear. A single juvenile polyp is believed to not increase the risk of cancer. This is based on a few studies with a limited number of patients. Nugent et al.^5^ studied the survival rate and cancer occurrence in 82 patients with solitary juvenile polyps and found no increased risk of cancer.

Several hypotheses exist for the mechanism by which juvenile polyps become malignant. The first hypothesis is that polyps follow a hamartoma-carcinoma sequence similar to adenoma-carcinoma. They grow more prominent, dysplasia develops, and eventually transforms into invasive carcinoma. The second theory is that cancer results not from the polyps themselves but from asymmetrical stem cell divisions, leading to altered stem cell lineage turnover rates and accelerated progression to cancer. The third hypothesis is based on the idea that juvenile polyps may develop foci of adenomas, which then can proceed to malignant degeneration.^6^

Juvenile polyps are most commonly diagnosed in children during their first decade of life. They are commonly found in the rectum and present with rectal bleeding. Less common presentations include abdominal pain, diarrhea, constipation, anemia, and prolapse of polyp through the rectum. In adults, rectal bleeding is the most common symptom, followed by abdominal pain and prolapse.^7^

It is important to distinguish patients with one juvenile polyp from patients with inherited polyposis syndromes. Patients with hamartomatous polyposis syndromes (HPS) have multiple polyps throughout the GI tract, extraintestinal findings (extraintestinal cancers, epistaxis, telangiectasias) and a high risk of cancer. Diagnosis of HPS is based on clinical criteria, and genetic testing is used to confirm the diagnosis. Juvenile polyposis syndrome is a rare hereditary disease characterized by multiple hamartomatous polyps throughout the gastrointestinal tract and the risk for colorectal and gastric cancer is increased. Ιt is important to distinguish the occurrence of one polyp from juvenile polyposis syndrome (JPs), which can be diagnosed based on the following clinical criteria: i) more than 5 juvenile polyps in the GI tract, ii) multiple juvenile polyps throughout the GI tract, or iii) one or more juvenile polyps together with a family history of JPs. Mutations are detected in SMAD4 and BMPR1A genes in 20-30% of cases.^8^ Peutz-Jeghers syndrome is characterized by intestinal polyps, especially in the small bowel and mucocutaneous pigmentations. *STK11* mutations are detected in more than 90% of patients with Peutz-Jeghers syndrome. *PTEN* hamartoma tumor syndrome includes Cowden Syndrome, Bannayan-Riley-Ruvalcaba Syndrome, *PTEN*-related Proteus syndrome, and Proteus-like Syndrome. Sporadic juvenile polyps of the colon are not associated with increased cancer risk and do not need genetic counseling. However, they may undergo dysplastic changes and, therefore, should be resected.^3^

**Figure 1:**
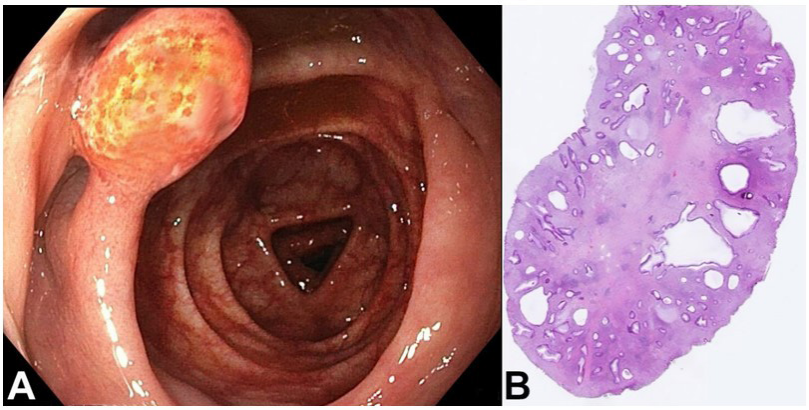
**A -** Endoscopic view of a pedunculated polyp in the transverse colon; **B -** photomicrograph of the polyp showing cystically dilated glands with chronic inflammatory cells.

Figure 1 refers to a 60‐year‐old woman who was submitted to a screening colonoscopy. She reported no previous endoscopic examination and had no family history of colorectal polyps or cancer. Endoscopic examination revealed a 15 mm pedunculated polyp (Figure 1A) in the transverse colon and was successfully removed endoscopically. The polyp’s histologic examination showed cystically dilated glands with chronic inflammatory cells, consistent with a juvenile polyp (Figure 1B). Finding a solitary polyp should prompt a search for such polyps at other locations in the gastrointestinal tract. Our patient underwent gastroscopy and capsule endoscopy, which revealed no other findings.
